# Distinct Metalloproteinase Expression and Functions in Systemic Sclerosis and Fibrosis: What We Know and the Potential for Intervention

**DOI:** 10.3389/fphys.2021.727451

**Published:** 2021-08-27

**Authors:** Edwin Leong, Michael Bezuhly, Jean S. Marshall

**Affiliations:** ^1^Department of Pathology, Dalhousie University, Halifax, NS, Canada; ^2^Department of Microbiology and Immunology, Dalhousie University, Halifax, NS, Canada; ^3^Department of Surgery, Dalhousie University, Halifax, NS, Canada

**Keywords:** fibrosis, systemic sclerosis, metalloproteinase, skin, lung, tissue inhibitors of metalloproteinase, inflammation

## Abstract

Systemic sclerosis (SSc) is a chronic debilitating idiopathic disorder, characterized by deposition of excessive extracellular matrix (ECM) proteins such as collagen which leads to fibrosis of the skin and other internal organs. During normal tissue repair and remodeling, the accumulation and turnover of ECM proteins are tightly regulated by the interaction of matrix metalloproteinases (MMPs) and endogenous tissue inhibitors of metalloproteinases (TIMPs). SSc is associated with dysregulation of the activity of these proteolytic and inhibitory proteins within the tissue microenvironment, tipping the balance toward fibrosis. The resultant ECM accumulation further perpetuates tissue stiffness and decreased function, contributing to poor clinical outcomes. Understanding the expression and function of these endogenous enzymes and inhibitors within specific tissues is therefore critical to the development of therapies for SSc. This brief review describes recent advances in our understanding of the functions and mechanisms of ECM remodeling by metalloproteinases and their inhibitors in the skin and lungs affected in SSc. It highlights recent progress on potential candidates for intervention and therapeutic approaches for treating SSc fibrosis.

## Introduction

Systemic sclerosis (SSc) is a complex chronic disease of unknown etiology, with external environmental factors, genetic predisposition, and epigenetic changes implicated in its development (Mora, 2009; Ramos et al., 2015; Angiolilli et al., 2018). SSc is characterized by persistent immune system activation, changes in vascularization, and excessive extracellular matrix (ECM) accumulation leading to fibrosis. There is considerable heterogeneity of clinical presentations in SSc patients, ranging from Raynaud’s phenomenon to severe fibrosis affecting critical organ function in severe cases (Sunderkötter and Riemekasten, 2006; Allanore et al., 2015; Varga et al., 2017). SSc can be classified as “limited” or “diffuse,” depending on the extent and progression of disease in the skin, with the latter involving more proximal skin locations and having increased impact on internal organ systems (Sobolewski et al., 2019). Uncontrolled fibrosis can impair organ function to the point of failure and even death (Varga and Abraham, 2007). Current treatments for SSc are limited with variable clinical response (Allanore et al., 2016; Kowal-Bielecka et al., 2017; Varga et al., 2017), and emerging therapeutics target fundamental fibrotic processes associated with SSc (Daoussis and Liossis, 2019). Metalloproteinases and their regulators play pivotal roles in mediating fibrosis, making them attractive therapeutic targets in SSc. Given their function in homeostasis, a thorough understanding of their roles in healthy and fibrotic tissues is required to develop safe and effective therapies targeting these pathways.

## Extracellular Matrix and Roles in Fibrosis

**FIGURE 1:**
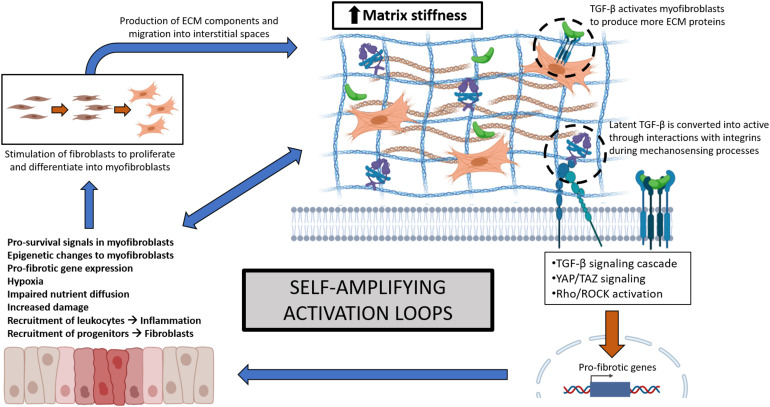
Self-amplifying activation loops involving multiple signaling elements during fibrosis, resulting from increased ECM accumulation and matrix stiffness. Increased tissue stiffness leads to reduced function, hypoxia, and triggers mechanosensory-related transcription factors, causing more damage, pro-fibrotic gene expression, and fibroblast activation and differentiation into myofibroblasts. Myofibroblasts contribute to ECM synthesis and accumulation and are supported by pro-survival signals and epigenetic changes to further enhance fibrosis. These self-amplifying mechanisms are conserved across different organ fibroses.

Early changes in ECM may provide means for better diagnostic accuracy in fibrotic disease (Abignano and Del Galdo, 2014; Burgstaller et al., 2017). However, clinical disease is often well established prior to diagnosis, reducing opportunities for early intervention. Increased ECM accumulation and matrix stiffness decreases tissue function, promoting further damage and perpetuating fibrosis (Lampi and Reinhart-King, 2018; Santos and Lagares, 2018). ECM accumulation and stiffness relay mechanical cues, initiate mechanosensory responses (Herrera et al., 2018; Tschumperlin et al., 2018), and provide major reservoirs for latent TGF-β (Annes et al., 2003; Xu et al., 2018) that is activated by conformational changes to further promote pro-fibrotic processes (Annes et al., 2004; Wipff et al., 2007). Myofibroblasts, a major source of ECM proteins, receive sustained survival and differentiation signals from matrix stiffness (Lagares et al., 2017; Van Caam et al., 2018). Further consequences include reduced nutrient diffusion, hypoxia, and epigenetic modifications of local cell populations (Beyer et al., 2009; Liu et al., 2010; Parker et al., 2014). These can change myofibroblast phenotypes by inducing transcription factor activity (Dupont et al., 2011; Dupont, 2019), specific microRNAs, and DNA methylation processes causing sustained cellular activation independent of the initial insult (Orphanides et al., 1997; Corpechot, 2002; Wang et al., 2006; Altorok et al., 2015). Together, these interactions drive multiple amplifying loops, further promoting cellular activation and enhanced ECM deposition (Figure 1). Thus, matrix stiffness caused by ECM accumulation needs to be tightly regulated to allow normal tissue remodeling and prevent pathologic fibrosis.

## Matrix Metalloproteinases and Their Inhibitors

Matrix stiffness is determined by a balance between ECM breakdown and accumulation, with the latter more significant in SSc (Varga and Bashey, 1995). Two main classes of mediators involved in this balance are matrix metalloproteinases (MMPs) and tissue inhibitors of metalloproteinases (TIMPs) (Nagase et al., 2006). Currently, there are 24 known MMPs in humans which are categorized based on their preferred substrates such as collagenases (MMP1, 8, 13) and gelatinases (MMP2, MMP9) (Loffek et al., 2011). TIMPs are the natural inhibitors of MMPs and classically inhibit ECM degradation (Brew and Nagase, 2010).

Current disease models suggest that increasing active MMPs and downregulating TIMPs would be beneficial to facilitate proper tissue remodeling and restoration of tissue homeostasis; however, MMPs and TIMPs have physiological implications beyond tissue remodeling (Giannandrea and Parks, 2014) including facilitating inflammation through cellular trafficking, immune cell activation, regulation of cytokines, chemokines, and activity of other MMPs (Nagase et al., 2006; Gaffney et al., 2015). The expression of MMPs is also cell- and tissue-specific and temporally regulated by immune and stromal cells, further adding to the complexity of this system (Pardo and Selman, 2006; Chuang et al., 2019). Finally, MMP functions depend on tissue and disease context (Loffek et al., 2011), and have both pro- and anti-fibrotic effects. To this end, global upregulation of MMPs and downregulation of TIMPs is ineffective in overcoming excessive ECM accumulation. Manipulation of this system requires an understanding of interactions between expressed MMPs and TIMPs within specific organs at specific stages of disease progression to be effective.

## Metalloproteinases in SSc Serum

Analysis of specific MMPs and TIMPs in the serum of SSc patients suggests possible influences on outcomes in different tissues. MMP7 was increased in SSc patients with considerably higher levels of observable lung fibrosis compared to healthy individuals (Moinzadeh et al., 2011). In contrast, MMP13 was reduced in serum from local and diffuse SSc patients compared to normal controls, with reductions reflecting greater tissue involvement and lung fibrosis (Asano et al., 2006a, b). Adding to the complexity, MMP9 was found increased in serum and tears of SSc patients along with increased TIMP-2, supporting a negative role for these mediators (Waszczykowska et al., 2020). Autoantibodies against MMP3 have been detected in SSc patients, and MMP3 considered beneficial as inhibition is suggested to reduce ECM degradation (Nishijima et al., 2004). It is evident that distinct MMPs may have multifaceted roles in ECM turnover and while MMP-TIMP expression in serum samples serve as potential biomarkers of SSc prognosis, involvement in organ fibrosis is likely far more complex than data on general upregulation or downregulation might suggest.

## Metalloproteinases in Skin Fibrosis

The MMP profile in skin tissue is limited and while well-studied in skin pathologies such as wound healing (Kähäri and Saarialho-Kere, 1997; Caley et al., 2015; Nguyen et al., 2016), less is known about their roles in fibrotic cutaneous disorders such as SSc. Skin involvement is present in both milder and severe forms of SSc and there is increased ECM deposition in the skin during fibrosis progression. A positive correlation is observed between presence of abnormal subtypes of collagen with disease stage and dermal thickness scores (Martin et al., 2012).

Significantly reduced levels of interstitial collagenase MMP1 expression have been found in biopsies of involved skin in a cohort of SSc patients (Frost et al., 2012). This was corroborated by other studies showing markedly reduced MMP1 gene expression and protein levels in dermal fibroblasts isolated from SSc patient skin. Several microRNAs increased in serum and skin of SSc patients were observed to downregulate MMP1 expression (Sing et al., 2012; Zhou et al., 2017). Peripheral blood mononuclear cells from SSc patients produced cytokines and growth factors in response to soluble type 1 collagen *in vitro* that elicited reduced MMP1 production by SSc dermal fibroblasts (Brown et al., 2012), pointing to additional forms of MMP1 regulation. CXCL17, a chemokine implicated in several tissue remodeling and antimicrobial processes, was found reduced in SSc skin and can regulate type 1 collagen expression by MMP1-associated mechanisms (Shimada et al., 2020). Local CXCL17 injections ameliorated fibrotic outcomes in a murine model of bleomycin (BLM)-induced skin fibrosis and CXCL17 treatment reduced type 1 collagen protein while increasing gene and protein expression of MMP1 in healthy human dermal fibroblasts.

MMP3, crucial for inhibition of pro-fibrotic α2-antiplasmin and ECM deposition, was decreased in SSc fibroblasts (Niwa et al., 2020). MMP3-stimulated SSc fibroblasts decreased α-SMA expression and type 1 collagen production. Given that collagenases function to breakdown ECM materials, in theory increasing skin-specific MMPs and reducing TIMPs should ameliorate fibrosis. However, this is not always the case. Increased MMP9 production was found in SSc patient dermal fibroblasts, positively correlating with the extent of SSc skin pathology (Kim et al., 2005) and likely due to MMP9 activities independent of ECM degradation, including regulation of inflammatory cytokines and growth factors (Van Den Steen et al., 2002). MMP9 degrades ECM but also other important non-cellular components that maintain effective barriers within the SSc skin microenvironment. A combination of barrier dysfunction and enhanced inflammatory and pro-fibrotic growth factors can enable an influx of immune cells, perpetuating inflammation and established fibrosis (Yu and Stamenkovic, 2000; Kim et al., 2005).

Along with dysregulation of MMPs, TIMPs are reported to be elevated in SSc skin. Fibroblasts derived from SSc biopsies demonstrated increased TIMP-1 compared to those from control skin (Kikuchi et al., 1997). Thus, reducing production of this inhibitor may be beneficial in SSc. The production of TIMP-1 by SSc dermal fibroblasts was reduced when treated with microRNA-29a, associated with increased MMP1 production (Ciechomska et al., 2014). Taken together, modulating the MMP-TIMP balance could potentially reverse the SSc fibroblast phenotype and offer therapeutic opportunities for treatment of skin fibrosis. However, considering the complex expression patterns of metalloproteinases and inhibitors in SSc patient skin, anti-fibrotic strategies involving ECM degradation by MMPs require further clinical study.

In non-SSc fibrotic conditions such as hypertrophic scarring, MMP3 and MMP8 are increased with TIMP members while other classical MMPs decreased. Several MMP-deficient strains of mice have been reported to have little change in phenotype, pointing toward functional redundancy between members of this family (Loffek et al., 2011). However, skin wound healing studies have shown MMP13 promotes rapid collagen remodeling and fibroblast survival, resulting in effective and scarless healing (Toriseva et al., 2007). MMP14-deficient mice demonstrate insufficient collagen turnover leading to connective tissue pathologies (Holmbeck et al., 1999), indicating that functions of MMP14 may not be readily compensated for by others. Using the BLM-induced skin fibrosis model (Yamamoto et al., 1999), MMP14-deficient mice exhibited significant accumulation of type 1 collagen in the skin but without changes to collagen synthesis (Zigrino et al., 2016), and fibroblasts from their skin showed impaired MMP2 activation. Therefore, MMP14 may be particularly important in degrading dermal ECM components and regulation of other MMPs. Taken together, these studies support studying these MMPs as targets for therapy in SSc skin.

## Metalloproteinases in Lung Fibrosis

The lungs are a major affected organ, where SSc manifests as interstitial lung disease (ILD) characterized by extensive fibrosis, leading to decreased functional capacity and even death (Cottin and Brown, 2019). Aberrant expression of MMPs have been reported, but with functions seemingly contrary to their classical ECM proteolytic activities. In multiple studies, elevated MMP7 serum levels have shown a negative correlation with lung function parameters such as forced vital capacity and associated with advanced stages of SSc. These observations suggest that MMP7 may, directly or indirectly, contribute to lung fibrosis, and may serve as a biomarker for SSc progression (Moinzadeh et al., 2011; Stock et al., 2019; Abo Gabal et al., 2020). In bronchoalveolar lavage fluid (BALF) of SSc patients with ILD, MMP9 levels were elevated compared to healthy controls, suggesting involvement in the persistence of inflammatory processes (Andersen et al., 2007). MMP12 is increased in serum and lung tissues of SSc patients with ILD and positively correlates with lung fibrosis severity. MMP12 expression was abundant in regions of thickened alveolar septa and expressed by alveolar macrophages and fibroblasts residing in fibrotic lung compartments (Manetti et al., 2012). A specific MMP12 polymorphism was also found to increase susceptibility to SSc and ILD in a cohort of SSc patients compared to healthy individuals (Manetti et al., 2010; Assassi et al., 2013). While MMP12 functions in SSc-ILD have not been defined, MMP12 downregulated expression of collagenase MMP13 in a *Schistosoma mansoni* Th2-driven model of lung fibrosis (Madala et al., 2010), providing a crucial example of MMP-dependent regulation of other metalloproteinases. Notably, serum MMP13 levels were lower in a subset of SSc patients compared to healthy controls, and reductions associated with significantly greater frequency of reduced vital capacity (Asano et al., 2006b). This suggests that MMP13 is associated with ECM remodeling and deficiency enhances lung fibrosis.

Matrix metalloproteinases have also been examined in idiopathic lung fibrosis (IPF)—another debilitating form of lung pathology arising from aberrant ECM deposition and remodeling. While there are differences between SSc-ILD and IPF, there are notable similarities particularly during the end-stages of the diseases (Herzog et al., 2014). These include signaling arising from injurious stimuli leading to pro-fibrotic events, and a pro-fibrotic phenotypic shift of pulmonary fibroblasts in response to ECM composition containing collagen, tenascin-C, and mechanical cues resulting from increased tissue stiffness (Brissett et al., 2012; Herzog et al., 2014).

Idiopathic lung fibrosis patient BALF show increased expression of MMPs, with MMP7, MMP8, and MMP9 predominating (Dancer et al., 2011). MMP7 is highly upregulated and activated in IPF patient lungs (Zuo et al., 2002; Fujishima et al., 2010), and is the most upregulated gene encoding for proteins involved in ECM remodeling. MMP7 can promote fibrosis in the early phases by inducing epithelial cell damage but have immunosuppressive and anti-fibrotic potential in persistent fibrotic lung environments in longer-term studies in MMP7-deficient animals (Li et al., 2002; Zuo et al., 2002; Manicone et al., 2009), highlighting disease stage influence on MMP7 involvement. MMP8 can act indirectly to enhance lung fibrosis by cleaving anti-inflammatory IL-10, an inhibitor of TGF-β, maintaining a pro-fibrotic environment (García-Prieto et al., 2010). Increased MMP8 expression was found in IPF patient BALF (Henry et al., 2002; Stijn et al., 2013; Craig et al., 2014) and others have shown MMP8 associated with significant reductions in CXCL10, reported to be anti-fibrotic in lungs (Marie Burdick et al., 1999; Tager et al., 2004; Jiang et al., 2010) by inhibiting lung fibroblast migration and differentiation into myofibroblasts (Craig et al., 2013). MMP3 is upregulated in IPF patients compared to healthy control lungs (Yamashita et al., 2011). MMP3-deficient mice exhibited reduced fibrosis in response to BLM and exogenous recombinant MMP3 promoted pro-fibrotic phenotypes. Further assessments found correlations between increased MMP3 and a collagenolytic by-product endostatin (Heljasvaara et al., 2005), which induces lung epithelial cell apoptosis (Richter et al., 2009) and thus promotes fibrosis.

Despite having pro-fibrotic roles in lungs as described above, specific MMPs show anti-fibrotic functions. Contrary to SSc-ILD, active MMP13 is increased in whole lung samples of IPF patients (Nkyimbeng et al., 2013). In IPF rodent models, MMP13-deficiency demonstrated exacerbated inflammatory responses to lung injury (Sen et al., 2010) and BLM-induced lung fibrosis, associated with increased leukocytic infiltration such as neutrophils (Nkyimbeng et al., 2013) and macrophages (Cabrera et al., 2019). Additionally, MMP13-deficient lung tissues had higher collagen staining and hydroxyproline content. Others showed MMP13-deficient mice having delayed resolution of lung fibrosis through decreased collagenolytic activity (Cabrera et al., 2019). Similarly, MMP19 is increased in IPF patients, and considered indicative of a tissue repair response (Yu et al., 2012). MMP19-deficient mice showed increased α-SMA staining and collagen deposition, possibly exerting its anti-fibrotic effects by mediating expression of cyclo-oxygenase 2 to suppress fibrosis by regulation of fibroblast proliferation and collagen synthesis (Wilborn et al., 1995; Pomianowska et al., 2014; Zhao et al., 2016). Thus, MMP19 may be an important anti-fibrotic metalloproteinase in the lungs through regulation of ECM deposition. MMP9 is classically pro-fibrotic (Kobayashi et al., 2014) and is increased in IPF patient BALF (Henry et al., 2002). However, significant anti-fibrotic activity was shown in a mouse model overexpressing human MMP9 in alveolar macrophages (Cabrera et al., 2007), potentially linked to inhibition of fibroblast growth (Valentinis et al., 1995). Appropriate regulation of cell growth, repair, and apoptosis were proposed to ameliorate the lung fibrosis in this model.

## MMPs in Other SSc-Associated Organ Systems

While cardiac, gastrointestinal (GI), and renal organ systems are involved in SSc, MMPs have not been directly studied in such clinical tissues. SSc cardiac complications range from ventricular dysfunction to infarction (Lambova, 2014), resulting in tissue damage and ensuing fibrosis (Tzelepis et al., 2007). In murine models, MMP-1 attenuated cardiac fibrosis development (Foronjy et al., 2008), but MMP-2 promoted ventricular remodeling, leading to failure-associated phenotypes (Bergman et al., 2007). MMP-12 induced production of TGF-β and platelet-derived growth factor, promoting accumulation of macrophage populations involved in fibrotic mechanisms (Stawski et al., 2014). GI manifestations are common in SSc patients, which can lead to fibrosis and ensuing motility and functional aberrations (Sallam et al., 2006; McFarlane et al., 2018). GI MMPs have not been directly studied in SSc, but have important functions in several intestinal inflammatory disorders (Medina and Radomski, 2006; O’Sullivan et al., 2015). While many point toward detrimental MMP functions during inflammation, one study has shown inhibited MMP synthesis can lead to excessive collagen deposition in fibrotic intestinal muscles (Bailey et al., 2012). Kidney involvement is also prevalent in SSc patients, leading to scleroderma renal crisis (SRC), promoted by interstitial fibrosis amongst other risk factors (Steen, 2014; Chrabaszcz et al., 2020). Direct MMP involvement in SSc-associated renal fibrosis has not been identified, but numerous studies imply a detrimental role of MMPs in early stages of kidney diseases with ECM-degradative features in later stages during scarring and fibrosis (Ke et al., 2017; Zakiyanov et al., 2019). Upregulated peptides in renal pathologies such as angiotensin-II promote renal fibrosis and regulate MMP expression, further affecting remodeling processes (Solini et al., 2011; Pushpakumar et al., 2013).

## MMPs as Therapeutic Targets for Fibrosis and SSc: Where Are We Currently?

As outlined above, MMPs demonstrate tissue-dependent and disease-specific expression and functions (summarized in Table 1). Clinical translation of broad-spectrum MMP inhibitors (MMPIs) have been largely unsuccessful despite success in some experimental models (de Meijer et al., 2010), likely due to beneficial MMP functions in homeostasis and immunity (Vandenbroucke and Libert, 2014). This is evidenced by discontinuation of poorly selective MMPIs in anti-cancer clinical trials due to adverse responses (Fields, 2019) and lack of MMP-based trials in SSc-ILD (Khanna et al., 2019). Cross-regulation of MMP members further add to the unpredictable impact of MMPIs. Novel methods for targeting metalloproteinases are necessary, with increased specificity and localized delivery to fibrotic sites. Developing selective synthetic peptides to block catalytic functions, targeting unique MMP domains to alter function, or manipulating endogenous MMP regulators like microRNAs represent some potential avenues of investigation (Li and Li, 2013; Mohan et al., 2016), serving as basis for novel proposed clinical trials in SSc skin (ClinicalTrials.gov identifier #NCT03740724). New treatment modalities should consider temporal regulation of MMPs and stages of disease in which they are active. Together, new classes of MMPIs with improved disease models will help our understanding of MMP profiles observed in fibrotic conditions such as in SSc.

**TABLE 1:** Summary table of selected MMPs with therapeutic potential for targeting in SSc skin and lungs to improve fibrosis and ECM accumulation.

MMP	Skin	Possible function / mechanism in the skin
Mmp1	SSc	Decreased; Collagen breakdown Frost et al. (2012); Sing et al. (2012); Zhou et al. (2017)
Mmp3	SSc	Decreased; Decreased α-SMA and type 1 collagen production Niwa et al. (2020)
Mmp9	SSc	Increased; Regulation of inflammatory cytokines and growth factors; Degrades non-cellular components of the skin barrier, leading to increased infiltration of inflammatory cell Kim et al. (2005)
Mmp13	Wound healing	Rapid collagen remodeling Toriseva et al. (2007)
Mmp14	BLM-induced skin fibrosis	Knockouts show significant type 1 collagen accumulation in skin, and impaired Mmp2 activation Holmbeck et al. (1999); Zigrino et al. (2016)

**MMP**	**Lung**	**Possible function / mechanism in the lungs**

Mmp3	IPF	Increased and produces endostatin, a by-product that increases lung epithelial cell death; Knockout mice have less lung fibrosis Heljasvaara et al. (2005); Richter et al. (2009); Yamashita et al. (2011)
Mmp7	SSc-ILD	Increased; Not defined Moinzadeh et al. (2011); Stock et al. (2019); Abo Gabal et al. (2020)
	IPF	Increased in BALF; Associated with epithelial cell damage and neutrophil recruitment in early stages, but associated with immunosuppressive dendritic cell recruitment in late stages Li et al. (2002); Zuo et al. (2002); Manicone et al. (2009); Fujishima et al. (2010)
Mmp8	IPF	Increased in BALF; Cleaves IL-10, a suppressor of TGF-β synthesis; decreased CXCL10 Wilborn et al. (1995); Henry et al. (2002); Tager et al. (2004); García-Prieto et al. (2010); Jiang et al. (2010); Dancer et al. (2011); Craig et al. (2013, 2014); Stijn et al. (2013)
Mmp9	SSc-ILD	Increased; Involved in persistence of inflammatory processes Andersen et al. (2007); Dancer et al. (2011)
	IPF	Increased in BALF; Overexpression in alveolar macrophages in mice showed anti-fibrotic activity *via* regulation of fibroblast growth Valentinis et al. (1995); Henry et al. (2002); Cabrera et al. (2007); Kobayashi et al. (2014)
Mmp12	SSc-ILD	Increased; Not defined Manetti et al. (2010, 2012); Assassi et al. (2013)
	*Schistosoma mansoni* lung fibrosis	Knockouts show increased fibrosis and decreased Mmp13 collagenase Madala et al. (2010)
Mmp13	IPF	Increased in whole lung; Knockouts have decreased collagenolytic activity and higher collagen levels Asano et al. (2006a); Sen et al. (2010); Nkyimbeng et al. (2013); Cabrera et al. (2019)
Mmp19	IPF	Increased in IPF patients, but indicative of tissue repair; Knockouts show increased collagen deposition, and may act through regulation of cyclo-oxygenase 2 expression Wilborn et al. (1995); Yu et al. (2012); Pomianowska et al. (2014); Zhao et al. (2016)

*Described roles in other similar types of pathology and disease are also given, with mechanisms that may be translatable to SSc pathology in the skin and lungs.*

From what is currently known regarding MMPs in skin and lungs of SSc patients, there are likely two main functions of MMPs, first as mediators of inflammation and tissue damage, and second as contributors to ECM remodeling. As previously mentioned, collagenases like MMP1 and MMP3 are reduced in settings of greater SSc skin fibrosis, and experimental models suggest that increasing their levels or activity may have beneficial effects. Conversely, inhibiting others like MMP9 could be beneficial to ameliorate pro-inflammatory triggers of SSc progression. The functions of MMP13 and MMP14 in promoting wound healing may also warrant further investigation in the context of SSc. In SSc-ILD, MMP7, 8, 9, and 12 seem to primarily exacerbate the disease rather than degrade ECM and may justify targeting them for local inhibition. Temporally regulating MMP expression or activity during different disease stages has merit, as demonstrated in the delayed resolution of IPF in MMP13-deficient. Future research should involve detailed human studies which consider disease stage and tissue site when examining the activities of specific MMPs. Without such information, there is a considerable risk of off-target effects of targeting MMPs.

## Concluding Remarks

Currently, therapeutics targeting regulation of MMP-TIMP balance in SSc have not successfully translated into clinical settings despite considerable effectiveness in pre-clinical models, with a lack of ongoing clinical trials. From previous and current studies, it is evident that MMPs and TIMPs have multiple roles which may either promote or inhibit fibrosis. Moreover, the complex cross-regulation of these molecules necessitates looking at the MMP-TIMP axis as a network rather than individually in the context of disease. The expression of MMPs and TIMPs are highly dependent on inciting stimulus, tissue site, and disease stage, amongst others. To this end, any therapeutic targeting metalloproteinases should be tailored to take these factors into account. Despite the challenges, there remains considerable potential in targeting specific MMP functions as part of a combinatorial treatment regime to target the inflammatory and/or tissue remodeling phases in fibrotic disease, improving the lives of thousands of patients each year.

## Author Contributions

EL drafted and wrote the manuscript and figures. MB and JM provided guidance, editing, and support with the manuscript. All authors provided conceptual input and reviewed the final manuscript.

## Conflict of Interest

The authors declare that the research was conducted in the absence of any commercial or financial relationships that could be construed as a potential conflict of interest.

## Publisher’s Note

All claims expressed in this article are solely those of the authors and do not necessarily represent those of their affiliated organizations, or those of the publisher, the editors and the reviewers. Any product that may be evaluated in this article, or claim that may be made by its manufacturer, is not guaranteed or endorsed by the publisher.
